# Pseudospin-induced chirality with staggered optical graphene

**DOI:** 10.1038/lsa.2016.94

**Published:** 2016-08-12

**Authors:** Jian-Long Liu, Wei-Min Ye, Shuang Zhang

**Affiliations:** 1 School of Physics and Astronomy, University of Birmingham, Birmingham B15 2TT, UK; 2 Department of Physics, Harbin Institute of Technology, Harbin 150001, China; 3 College of Optoelectronic Science and Engineering, National University of Defense Technology, Changsha 410073, China

**Keywords:** angular momentum, chirality, pseudospin, staggered optical graphene

## Abstract

Pseudospin has an important role in understanding many interesting physical phenomena that are associated with two-dimensional materials such as graphene. Pseudospin has been proposed to be directly related to angular momentum, and orbital angular momentum was recently experimentally demonstrated to be an intrinsic property of pseudospin in a photonic honeycomb lattice. However, in photonics, the interaction between spin and pseudospin for light has not been investigated. In this letter, we propose that in an optical analog of staggered graphene (that is, a photonic honeycomb lattice waveguide with in-plane inversion symmetry breaking), the pseudospin mode can strongly couple to the spin of an optical beam that is incident in certain directions. The spin–pseudospin coupling that is caused by the spin–orbit conversion in the scattering process induces a strong optical chiral effect for the transmitted optical beam. Spin–pseudospin coupling of light opens the door to the design of pseudospin-mediated spin or valley-selective photonic devices.

## Introduction

Graphene and graphene-like two-dimensional (2D) materials have attracted significant research interest in recent years^1, 2, 3, 4, 5^. Particular attention has been paid to their unique electronic band structures, which exhibit linear dispersion near the Dirac points at the corners of the Brillouin zone. This unusual band feature gives rise to many interesting electron-transport properties, including the quantum hall effect^1, 2^, Zitterbewegung^3^ and the Klein paradox^4^. In single-layer graphene that is deposited on a substrate such as boron nitride and silicon carbide, the inversion symmetry between the two sublattices is broken. This symmetry breaking opens up gaps at the Dirac points, which leads to an interesting optical selection rule at different valleys^6, 7, 8, 9, 10^. Specifically, the optical transitions at the two valleys can be excited by light of different circular polarizations or spins^11, 12, 13^. Inversion symmetry breaking also leads to lift of the degeneracy between the two sublattice pseudospins. In contrast, in an ideal graphene without staggering potential, the pseudospin is considered to be unmeasurable and cannot interact with any magnetic field, even though it has been predicted to exhibit real orbital angular momentum (OAM)^14, 15^.

Recently, 2D photonic crystals with hexagonal lattices have provided a successful platform for demonstrating optical analogs of some of the interesting electronic properties of graphene^16^. Recent advances in this field include the demonstration of an optical spin Hall effect^17^, the discovery of unconventional edge states^18^, the demonstration of photonic Floquet topological insulators^19^ and several other interesting optical phenomena which are based on metasurfaces and photonic and plasmonic crystals^20, 21, 22^. Artificial optical graphene has been used to demonstrate that pseudospin is a measurable physical quantity. In particular, multiple-beam interference, which is carefully aligned with the honeycomb lattice, has been employed to excite the pseudospin modes, which were shown to exhibit OAM^23^. In this paper, we show that in a staggered optical graphene (SOG) in which the inversion symmetry between the two sublattices is broken, the pseudospin optical modes can be directly excited by a single circularly polarized beam with the aid of spin–pseudospin coupling, which leads to strong optical activity for an incident wave with in-plane wave-vector that matches the location of the Dirac points of the lattice in the reciprocal plane. Therefore, SOG not only represents a facile, lossless approach for achieving strong optical chirality, which normally requires complex three-dimensional metallic chiral structures, but also provides a platform for investigating the extraordinary phenomena that are associated with the pseudospin state of light in a honeycomb lattice.

## Materials and methods

In previous studies, artificial photonic graphenes have been developed using coupled optical resonators or waveguides, where each resonator or waveguide serves as the optical analog of a carbon atom in graphene. As a result, the coupling between the adjacent resonators or waveguides can be treated as photon hopping in a similar manner as electron hopping in their electronic counterparts. Under these conditions, the tight-binding approximation and Hamiltonian approach can be conveniently adopted^14, 17, 23, 24^. Here, without resorting to the tight-binding conditions, we show that OAM is an intrinsic property of pseudospin in a photonic honeycomb lattice. The derivation is purely based on symmetry operations; the details are given below.

**Figure 1:**
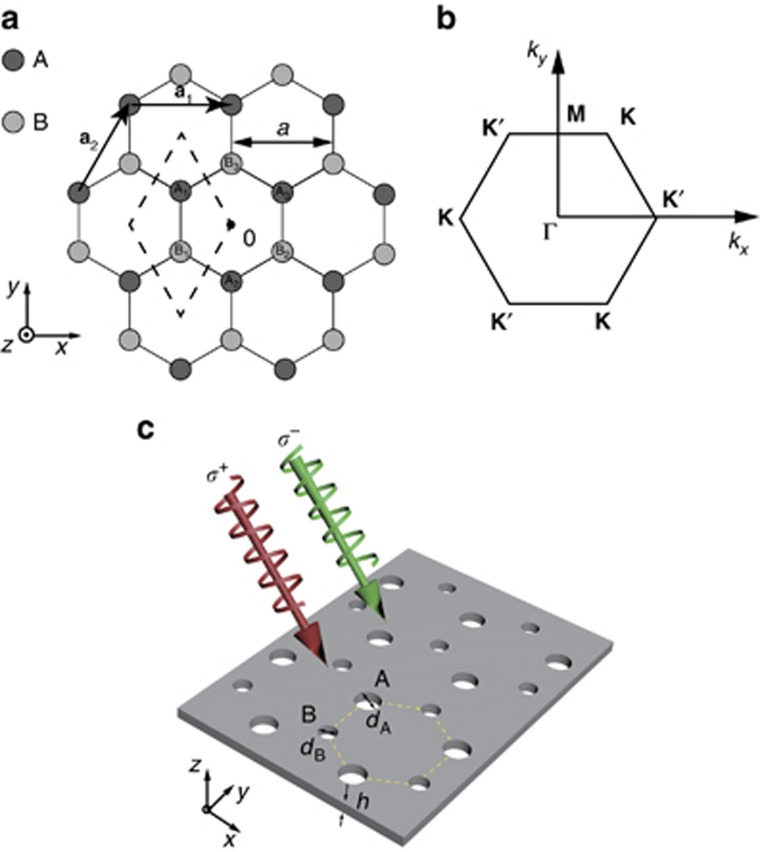
Illustration of the honeycomb lattice. (**a**) A honeycomb lattice with a lattice constant *a* and lattice vectors 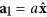 and 
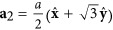
. (**b**) The first Brillouin zone in the reciprocal space with the positions of **K** and **K**′ indicated. (**c**) Schematic illustration of an artificial SOG based on a 2D photonic-crystal slab. The slab is illuminated by a plane wave with circular polarizations.

Figure 1a shows a schematic representation of a 2D honeycomb lattice for investigating spin–pseudospin coupling. The primitive lattice vectors of the honeycomb lattice are 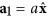 and 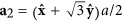. We consider the eigenmode of the 2D honeycomb lattice with an in-plane vector 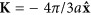 (Figure 1b). The in-plane vector **K** is unchanged by the rotation operation 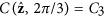 with respect to the center of the hexagon cell 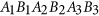. The action of the *C*_3_ rotational operator on the eigenmode at the **K** point can be written as^25^


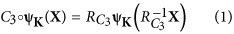


where


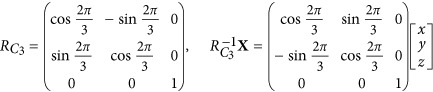


From Figure 1a, we can write


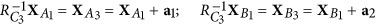


where 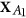 and 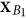 are the positions of element *A*_1_ and *B*_1_, respectively, in a hexagonal cell. Thus, Equation (1) can be rewritten as


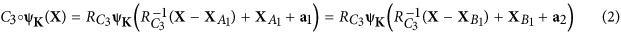


Because the lattice has translational invariance, the eigenmode of the 2D honeycomb lattice satisfies


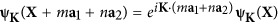


Because the in-plane vector **K** is unchanged by the rotation operation *C*_*3*_, it can be deduced from Equation (2) that


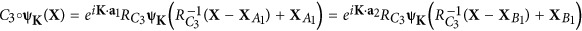


which can be rewritten as


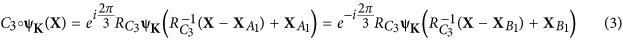


On the basis of Equation (1), Equation (3) can be rewritten using the rotation operation 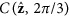 with respect to the centers of elements *A*_1_ and *B*_1_ as


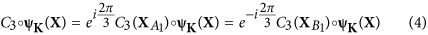


For honeycomb structures with *C*_3_ rotational symmetry, the eigenmode at the **K** point satisfies


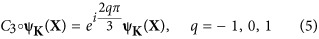


The OAM of an optical mode is directly related to its angular phase distribution around a certain point. Alternatively, it is manifested as the phase that is acquired by the mode when it is rotated by certain angles (depending on the rotational symmetry of the system). Equation (5) shows that rotating the eigenmode around the center of the hexagon by 120° introduces phase terms of −2*π*/3, 0 and 2*π*/3 for *q*=−1, 0 and +1, respectively, which correspond to OAMs of 1, 0 or −1, respectively. By combining Equations (4) and (5), it can be deduced that


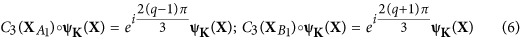


From Equation (6), we can deduce that the eigenmode with *q*=−1 has OAMs of −1 at sublattice A and 0 at sublattice B; the eigenmode with *q*=0 has OAMs of 1 at sublattice A and −1 at sublattice B; and the eigenmode with *q*=1 has OAMs of 0 at sublattice A and 1 at sublattice B. Because of the high spatial symmetry of the *q*=0 mode, it cannot be excited by an incident plane wave. We therefore focus on the two modes with *q*=±1. Similar to graphene, the eigenmodes of *q*=1 and −1 can be endowed with pseudospins 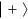 and 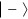, respectively, which possess OAMs of (0, 1) and (−1, 0) at sublattices (A and B), respectively. Because of the spin–orbit conversion of light during the scattering process, the eigenmodes with different pseudospins can be selectively excited by external circularly polarized light of different handednesses.

## Results and discussion

**Figure 2:**
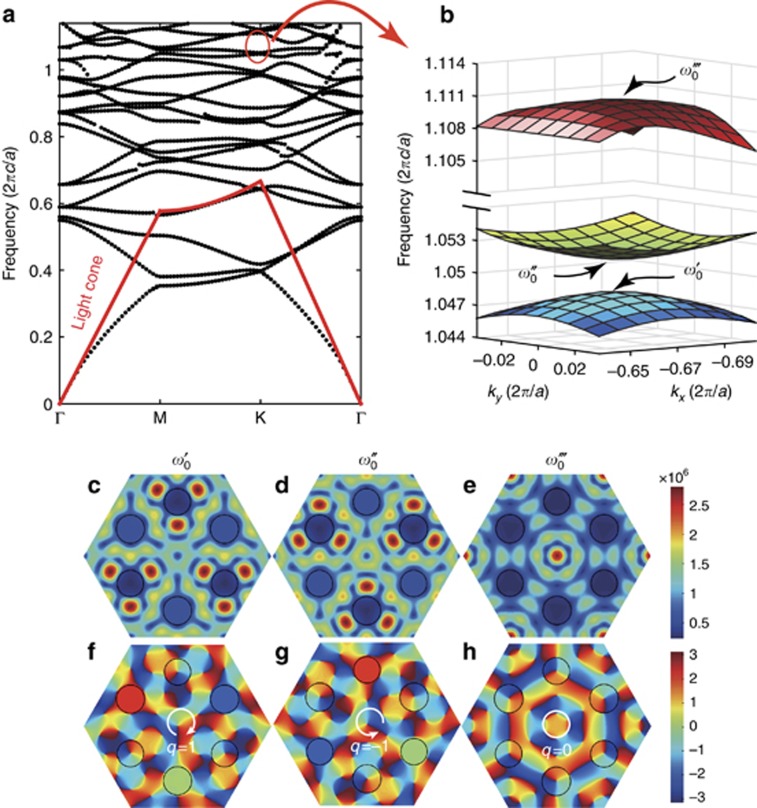
Eigenmodes supported by the 2D photonic lattice. (**a**) Band structure for the even modes in the SOG. (**b**) Dispersion relation in the vicinity of the **K** point 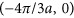. (**c**–**e**) Amplitude (|*H*_z_|) distributions on the symmetric plane in the *z* direction for the eigenmodes at the **K** point for *q*=1 (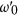
= 1.048 × 2*πc*/*a*), *q*=−1 (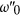
=1.052 × 2*πc*/*a*) and *q*=0 (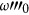
= 1.110 × 2*πc*/*a*), respectively. (**f–h**) Phase distributions (arg(*H*_z_)) of the three modes that correspond to *q*=+1, −1 and 0, respectively.

To confirm the theoretical analysis that was presented above, we numerically study a realistic SOG that is based on a photonic-crystal slab of thickness *h*. Figure 1c shows a schematic illustration of the SOG. The photonic lattice consists of two sets of circular apertures with diameters *d*_A_ and *d*_B_. The simulation uses a dielectric slab with a refractive index *n*=3 (which corresponds to AlAs at a wavelength ~800 nm), and the other geometric parameters are set as *h*=0.2*a*, *d*_A_=0.30*a* and *d*_B_=0.28*a*. The band diagram for this structure is plotted in Figure 2a. The dispersion relation of the eigenmodes near one **K** point 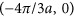 is calculated using the commercial FEM software COMSOL and is plotted in Figure 2b. The eigenfrequencies (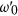
= 1.048 × 2*πc*/*a* and 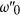
=1.052 × 2*πc*/*a*) at the **K** point lie inside the light cone, which means that the eigenmodes of the SOG can couple directly with external light fields. Figure 2c and 2d shows the field distributions (|*H*_z_|) on the symmetric plane in the *z* direction for the two eigenmodes with frequencies of 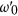 and 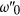, respectively. As expected, the field distributions show a threefold rotational symmetry. The phase patterns (arg(*H*_z_)) of the modes that were obtained from the simulations are shown in Figure 2f and 2g, respectively. Figure 2f shows that phase vortices are located at lattice B and are accompanied by an opposite phase vortex (*q*=1) located at the center of the hexagon cell. For the eigenmode with *q*=−1 (Figure 2g), the opposite phase vortices are located only at lattice A. We also calculate the amplitude and phase pattern of an eigenmode with *q*=0, which are shown in Figure 2e and 2h, respectively. The eigenfrequency is 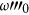
= 1.110 × 2*πc*/*a*, which is relatively far from the resonance frequencies of the other two modes. Opposite phase vortices are located at lattices A and B, while no phase vortex is present at the center of the hexagon cell (which corresponds to *q*=0). The simulation results are consistent with the theoretical analysis.

**Figure 3:**
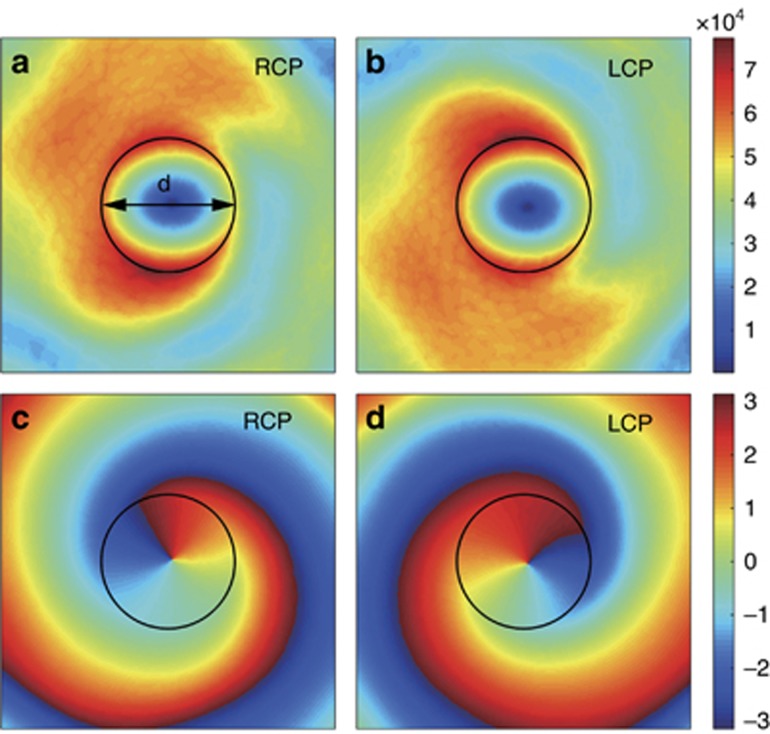
Conversion of the SAM of incident light into OAM of the guided mode through scattering by an individual circular aperture. (**a**, **b**) Simulated field amplitude (|*H*_z_|) distribution produced by incident RCP **a** and LCP **b** beams. (**c**, **d**) The phase (arg(*H*_z_)) distributions that are produced by RCP **c** and LCP **d** incident waves. In the simulation, the diameter of the circular aperture *d*=0.28*a*.

We propose to use a circularly polarized optical beam to selectively excite the pseudospins in the SOG. The spin angular momentum of the incident light can be converted to OAM by light scattering upon subwavelength objects^26, 27, 28^. We use a numerical simulation to check the spin–orbit AM conversion. In the simulation, a dielectric slab with a single air hole is illuminated by right- and left-circular polarized light (RCP/LCP). The diameter of the hole is 0.28*a*, and the other parameters are kept the same as in the mode calculation in the previous sections. The frequency of the incident light is s 1.048 × 2*πc*/*a*, which is the same as the eigenfrequency 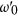 in the mode calculation. The incidence is inclined with the *k* vector 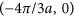. The simulation results, including the amplitude (|*H*_z_|) and phase (arg(*H*_z_)) distributions on the symmetric plane in the *z* direction, are shown in Figure 3a–3d, respectively. For an incident plane wave with circular polarization at normal incidence onto a lossless circular object, no transfer of the angular momentum occurs between the light and the object; the angular momentum must be conserved. Consequently, the wave scattered into the guided mode has an exact OAM of 1 or −1 because the guided mode is linearly polarized and does not carry spin angular momentum. In our case, however, the incidence is inclined (almost 40°), so the spin of the light that is projected onto the plane is less than 1. This leads to a non-uniform angular distribution of the field amplitude around the aperture as shown in Figure 3a and 3b. Nonetheless, the phase distributions clearly exhibit opposite vorticities that are located at the center of the hole (Figure 3c and 3d). This spin–orbit AM conversion provides a bridge that links the spin of the incident light and the pseudospins of the SOG.

**Figure 4:**
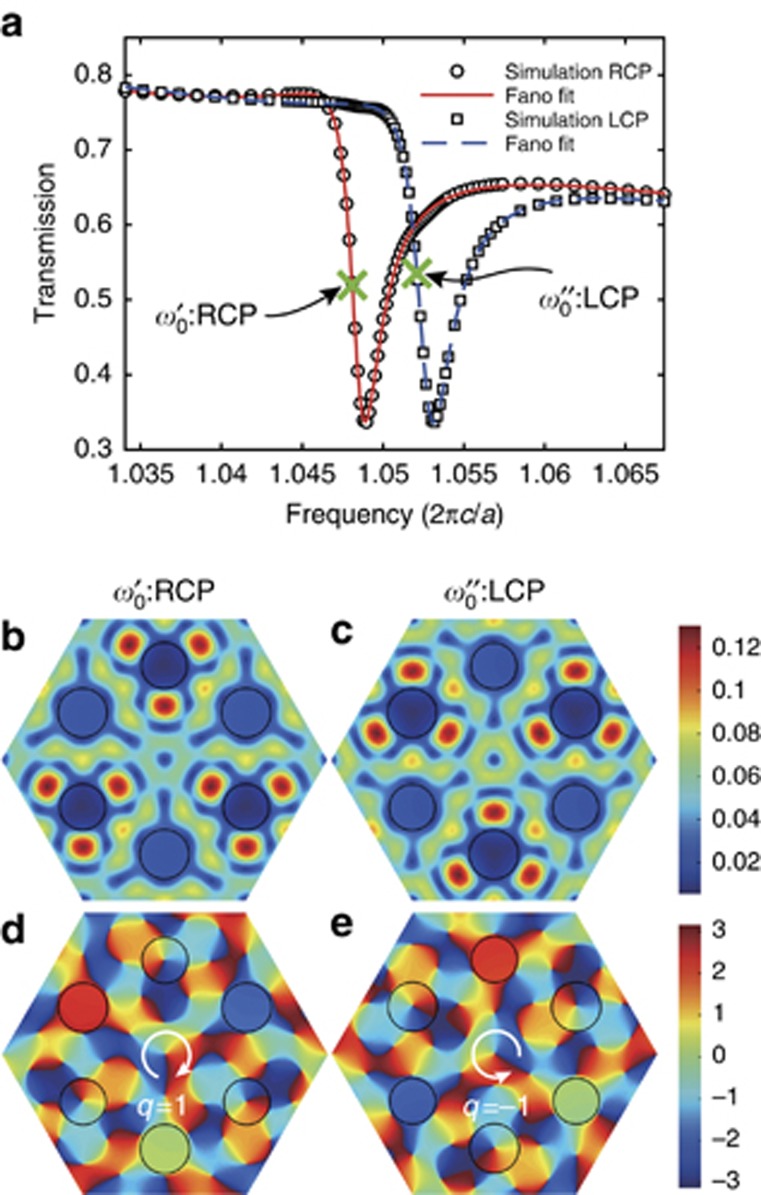
Chiral response of the 2D photonic-crystal through spin–pseudospin coupling. (**a**) Simulated and Fano-fitted spectra of zero-order transmission for the SOG. The structure is illuminated by RCP and LCP light beams with an in-plane wave vector along the **K** point. The fitting parameters are *C*_1_=0.049, *α*=2.77, Γ=2.2 × 10^−3^, *ω*_0_=1.048, *C*_2_=3.04 and *ω_d_*=0.971 for the RCP incidence and *C*_1_=0.041, *α*=2.98, Γ=2.6 × 10^−3^, *ω*_0_=1.053, *C*_2_=3.39 and *ω_d_*=0.978 for the LCP incidence. The eigenfrequencies of the pseudospin modes are indicated by the cross symbols. (**b**, **c**) Amplitudes (|*H*_z_|) on the symmetric plane of the slab in the *z* direction for RCP **b** and LCP **c** incidence at their respective eigenfrequencies. **(d**, **e**) The corresponding phase distributions of *H*_z_.

To demonstrate the spin–pseudospin coupling and the selective excitation of pseudospins, simulations are carried out to calculate the transmission spectrum of the SOG. The incident circularly polarized light with the fixed in-plane wave vector **K** 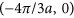 illuminates the photonic slab as shown in Figure 1c. All of the geometric parameters are the same as in Figure 2b. The zero-order transmittance is measured and shown in Figure 4a. There is a significant difference in transmission between the RCP and LCP incidences near the two eigenfrequencies, which confirms the presence of very strong optical chirality. The key reason for this transmission difference is the broken inversion symmetry of the SOG structure. Because the inversion symmetry is broken, the bandgap at the Dirac point is open and the two pseudospin states have different eigenfrequencies. Thus, when the incident RCP and LCP light couples with the pseudospins, the resonance peaks in the transmission spectra between the two circular polarizations are different. In our setting, the photonic-crystal slab is not an ideal 2D sheet because of the finite thickness in the *z* direction. An analysis is performed based on Fano line shapes to describe the interference between the scattered fields from the A and B sublattices and the plane slab^29^. The fitting curve is expressed as


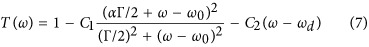


where *ω*_0_ is the resonant frequency, Γ is the linewidth (full-width at half-maximum), *α* is the Breit–Wigner–Fano coupling coefficient^30^ and *C*_1_, *C*_2_ and *ω*_*d*_ are three coefficients to be fitted. The third term on the right-hand side of Equation (7) represents the contribution of the direct transmission of a homogeneous slab with an effective refractive index. This term represents the background of the spectrum in Figure 4a. Because the frequency band we are concerned with is very narrow, the background spectrum can assume a linear form that is fitted by *C*_2_ and *ω*_*d*_ within this narrow range. Figure 4a shows the Fano line-shape fitted curves and the simulation data. The curves all agree well with the simulation data, and the fitted resonant frequencies are consistent with the simulated eigenfrequencies in the band calculation that is shown in Figure 2b.

To verify the contribution of the spin–pseudospin coupling to the transmission spectrum, we examine the field distributions when the crystal is illuminated by RCP and LCP light. As in Figure 2c, 2d, 2f and 2g, we extract the amplitude and phase of the magnetic field on the symmetric plane in the *z* direction. The frequencies of the incident light are fixed at the eigenfrequencies at the **K** point. The results are shown in Figure 4b–4e. The amplitude and phase patterns show good agreement with those of the eigenmodes that are shown in Figure 2, which confirms that the incident circularly polarized beam can excite pseudospins with matched handedness.

In the above simulations, we have used a free-standing 2D photonic-crystal with no added substrate. Free-standing 2D photonic-crystal membranes based on silicon or other semiconductor materials have been demonstrated by many groups^31, 32, 33^. Therefore, the symmetric configuration that is investigated in our paper can be fabricated without posing significant technical challenges. On the other hand, the presence of a substrate does affect the performance. If a substrate (for example, glass) is added, the *Q*-values of the leaky modes will decrease, and the contrast between the RCP and LCP transmission is expected to decrease.

## Conclusions

We studied spin–pseudospin coupling in a SOG structure. We predict and numerically demonstrate that the two pseudospin states at the Dirac point of the SOG can be directly excited by external circularly polarized light with opposite handedness. As a result, we show that the transmission spectrum of the lattice exhibits strong chirality, which arises from the coupling between the spin and the intrinsic handedness of the pseudospin. Because of the inversion symmetry of the reciprocal space, the spin–orbit interaction from the pseudospin leads to coupling of the spin and valley degrees of freedom, which makes it possible to selectively choose the spin of the incident light at different valleys. Because this spin–valley coupling occurs inside the light cone, this study may also provide opportunities to construct valley-dependent circularly polarized light emitters or generators.

## References

[bib1] Zhang YB, Tan YW, Stormer HL, Kim P. Experimental observation of the quantum Hall effect and Berry's phase in graphene. Nature 2005; 438: 201–204. 16281031 10.1038/nature04235

[bib2] Novoselov KS, Geim AK, Morozov SV, Jiang D, Katsnelson MI et al. Two-dimensional gas of massless Dirac fermions in graphene. Nature 2005; 438: 197–200. 16281030 10.1038/nature04233

[bib3] Katsnelson MI. Zitterbewegung, chirality, and minimal conductivity in graphene. Eur Phys J B 2006; 51: 157–160.

[bib4] Katsnelson MI, Novoselov KS, Geim AK. Chiral tunnelling and the Klein paradox in graphene. Nat Phys 2006; 2: 620–625.

[bib5] Castro Neto AH, Guinea F, Peres NMR, Novoselov KS, Geim AK. The electronic properties of graphene. Rev Mod Phys 2009; 81: 109–162.

[bib6] Yao W, Xiao D, Niu Q. Valley-dependent optoelectronics from inversion symmetry breaking. Phys Rev B 2008; 77: 235406.

[bib7] Xiao D, Liu GB, Feng WX, Xu XD, Yao W. Coupled spin and valley physics in monolayers of MoS_2_ and other group-VI dichalcogenides. Phys Rev Lett 2012; 108: 196802. 23003071 10.1103/PhysRevLett.108.196802

[bib8] Cao T, Wang G, Han WP, Ye HQ, Zhu CR et al. Valley-selective circular dichroism of monolayer molybdenum disulphide. Nat Commun 2012; 3: 887. 22673914 10.1038/ncomms1882 PMC3621397

[bib9] Zeng HL, Dai JF, Yao W, Xiao D, Cui XD. Valley polarization in MoS_2_ monolayers by optical pumping. Nat Nanotechnol 2012; 7: 490–493. 22706701 10.1038/nnano.2012.95

[bib10] Sie EJ, McIver JW, Lee YH, Fu L, Kong J et al. Valley-selective optical Stark effect in monolayer WS_2_. Nat Mater 2015; 14: 290–294. 25502098 10.1038/nmat4156

[bib11] Mak KF, McGill KL, Park J, McEuen PL. The valley Hall effect in MoS_2_ transistors. Science 2014; 344: 1489–1492. 24970080 10.1126/science.1250140

[bib12] Xu XD, Yao W, Xiao D, Heinz TF. Spin and pseudospins in layered transition metal dichalcogenides. Nat Phys 2014; 10: 343–350.

[bib13] Srivastava A, Sidler M, Allain AV, Lembke DS, Kis A et al. Valley Zeeman effect in elementary optical excitations of monolayer WSe_2_. Nat Phys 2015; 11: 141–147.

[bib14] Mecklenburg M, Regan BC. Spin and the honeycomb lattice: lessons from graphene. Phys Rev Lett 2011; 106: 116803. 21469887 10.1103/PhysRevLett.106.116803

[bib15] Trushin M, Schliemann J. Pseudospin in optical and transport properties of graphene. Phys Rev Lett 2011; 107: 156801. 22107311 10.1103/PhysRevLett.107.156801

[bib16] Xia FN, Wang H, Xiao D, Dubey M, Ramasubramaniam A. Two-dimensional material nanophotonics. Nat Photonics 2014; 8: 899–907.

[bib17] Nalitov AV, Malpuech G, Terças H, Solnyshkov DD. Spin-orbit coupling and the optical spin hall effect in photonic graphene. Phys Rev Lett 2015; 114: 026803. 25635557 10.1103/PhysRevLett.114.026803

[bib18] Plotnik Y, Rechtsman MC, Song DH, Heinrich M, Zeuner JM et al. Observation of unconventional edge states in ‘photonic graphene’. Nat Mater 2014; 13: 57–62. 24193661 10.1038/nmat3783

[bib19] Rechtsman MC, Zeuner JM, Plotnik Y, Lumer Y, Podolsky D et al. Photonic Floquet topological insulators. Nature 2013; 496: 196–200. 23579677 10.1038/nature12066

[bib20] Chan CT, Hang ZH, Huang X. Dirac dispersion in two-dimensional photonic crystals. Adv Optoelectron 2012; 2012: 313984.

[bib21] Weick G, Woollacott C, Barnes WL, Hess O, Mariani E. Dirac-like plasmons in honeycomb lattices of metallic nanoparticles. Phys Rev Lett 2013; 110: 106801. 23521276 10.1103/PhysRevLett.110.106801

[bib22] Shitrit N, Yulevich I, Maguid E, Ozeri D, Veksler D et al. Spin-optical metamaterial route to spin-controlled photonics. Science 2013; 340: 724–726. 23661756 10.1126/science.1234892

[bib23] Song DH, Paltoglou V, Liu S, Zhu Y, Gallardo D et al. Unveiling pseudospin and angular momentum in photonic graphene. Nat Commun 2015; 6: 6272. 25687645 10.1038/ncomms7272

[bib24] Koshino M, Morimoto T, Sato M. Topological zero modes and Dirac points protected by spatial symmetry and chiral symmetry. Phys Rev B 2014; 90: 115207.

[bib25] Sakoda K. Optical Properties of Photonic Crystals. 2nd edn. Berlin: Springer-Verlag; 2005.

[bib26] Vuong LT, Adam AJL, Brok JM, Planken PCM, Urbach HP. Electromagnetic spin-orbit interactions via scattering of subwavelength apertures. Phys Rev Lett 2010; 104: 083903. 20366932 10.1103/PhysRevLett.104.083903

[bib27] Bliokh KY, Ostrovskaya EA, Alonso MA, Rodriguez-Herrera OG, Lara D et al. Spin-to-orbital angular momentum conversion in focusing, scattering, and imaging systems. Opt Express 2011; 19: 26132–26149. 22274201 10.1364/OE.19.026132

[bib28] Haefner D, Sukhov S, Dogariu A. Spin hall effect of light in spherical geometry. Phys Rev Lett 2009; 102: 123903. 19392279 10.1103/PhysRevLett.102.123903

[bib29] Fan S, Joannopoulos J. Analysis of guided resonances in photonic crystal slabs. Phys Rev B 2002; 65: 235112.

[bib30] Scott JF. Soft-mode spectroscopy: experimental studies of structural phase transitions. Rev Mod Phys 1974; 46: 83–128.

[bib31] Altug H, Vučković J. Two-dimensional coupled photonic crystal resonator arrays. App Phys Lett 2004; 84: 161–163.

[bib32] Song BS, Noda S, Asano T, Akahane Y. Ultra-high-*Q* photonic double-heterostructure nanocavity. Nat Mater 2005; 4: 207–210.

[bib33] Eichenfield M, Chan J, Camacho RM, Vahala KJ, Painter O. Optomechanical crystals. Nature 2009; 462: 78–82. 19838165 10.1038/nature08524

